# Nicotine Dependence as a Mediator of Project EX’s Effects to Reduce Tobacco Use in Scholars

**DOI:** 10.3389/fpsyg.2016.01207

**Published:** 2016-08-12

**Authors:** María T. Gonzálvez, José P. Espada, Mireia Orgilés, Alexandra Morales, Steve Sussman

**Affiliations:** ^1^Department of Health Psychology, Miguel Hernández UniversityElche, Spain; ^2^Department of Preventive Medicine, University of Southern California, Los AngelesCA, USA

**Keywords:** nicotine dependence, Project EX, tobacco, cessation, prevention, school-based, adolescents, mediation analysis

## Abstract

In Spain, 44% of 14–18-year-olds have smoked, and 12.5% have smoked cigarettes in the last 30 days. Nicotine is one of the most addictive substances, and can lead to serious addiction in adulthood with adverse consequences to one’s health. School plays a relevant role in health promotion and preventing risk behaviors such as tobacco consumption. Despite the fact that some school-based tobacco cessation and prevention interventions prove to be effective for their purposes, there is a lack of understanding as to why these programs succeed or fail. This longitudinal study aims to test the nicotine dependence (ND) as a mediator of Project EX’s effect – a tobacco-use cessation program developed for high school youth to reduce tobacco consumption in scholars. Six high schools located in the Mediterranean coast were randomized for the participation of the program (Spanish version of Project EX) or a waiting-list group with baseline, immediate-posttest, and 12-month follow-up assessments. At baseline, 1,546 adolescents aged 14–21 years old (mean age: 15.28; *SD* = 1.20; 46% were women) were evaluated by self-administered tests on tobacco consumption and ND. A biomarker of smoke inhalation – a measurement of exhaled carbon monoxide (ECM) – was used. Participants who were smokers (*N* = 501; 32%) were selected for this study. Mediation analyses were conducted using the PROCESS v2.12 macro for Windows. The significant criterion was *p* ≤ 0.05, and 5,000 samples were used for bias-corrected bootstrap confidence intervals. Results indicated that Project EX indirectly decreased the number of cigarettes smoked in the last month, the number of cigarettes smoked within the last 7 days, the number of daily cigarettes, and ECM level at 12-month follow up through decreasing the level of ND in the short-term. This is the first Spanish study that explores ND as a mediator of the long-term efficacy of Project EX to reduce tobacco consumption in adolescents. Results suggest that interventions that reduce ND at short-term are more likely to be successful to decrease tobacco use at long-term.

## Introduction

Tobacco consumption is the leading preventive cause of disease and early death in developed countries (World Health Organization [WHO], 2014). Teens who experiment with tobacco consumption later become regular users and progress to regular use of other more harmful substances (Botvin and Griffin, 2007). In recent years there has been a growing interest in interventions for teen smoking cessation, since this population comprises the most vulnerable group, as it is at adolescence when addiction starts (Abad and Ruiz-Juan, 2015).

In Spain, tobacco is the second most consumed addictive substance, according to the latest data from the National Drug Plan (Spanish Drugs Observatory, 2015). It is estimated that 44% of adolescents have smoked on at least one occasion, 35% have smoked in the last year, and 12.5% have smoked cigarettes in the last 30 days. Motivations that lead adolescents to smoke are diverse and include the interaction of genetic and environmental factors that favor the initiation, experimentation, and consolidation of a level of nicotine dependence (ND; Cogollo-Milanés and de La Hoz-Restrepo, 2010). Sensation seeking, low risk perception, peer acceptance, and smoking social component favor adolescents risk behaviors (Latorre et al., 2014; Cantó et al., 2015). Also, adolescents who start smoking at a young age have a higher ND (Balboa et al., 2011; Dierker et al., 2015), establishing it as the main factor maintainer of smoking behavior (Villalobos et al., 2015).

Nicotine dependence is considered a construct which brings cognitive, behavioral, and physiological symptoms that characterize compulsive consumption (Villalobos et al., 2015). Dependence level can be considered a continuous variable, scoring from 0 to 19, where scores from 0 to 2 is considered low dependence, scores 3–4 is low dependence, 5 is medium dependence, scores 6–7 is high dependence, and from 8 to 19 very high dependence (Fagerström, 1978; Prokhorov et al., 2000). In turn, these dependence levels are established as a risk factor in the onset of depressive symptoms and negative moods (Gonzálvez et al., 2015), as well as schizophrenia and alcoholism (Becoña and Míguez, 2004), so it is necessary to design interventions to prevent tobacco consumption.

Project EX (Sussman et al., 2004) is an empirically validated school-based smoking-cessation intervention for adolescents developed in the United States (California). The intervention focuses on personal skills (such as assertiveness training), coping withdrawal, and motivational factors. The Project EX clinic version shows consistently positive effects through several controlled studies in the U.S. to prevent and reduce tobacco consumption in adolescents. In order to evaluate the efficacy of the program in other cultures the program was implemented and evaluated in several other countries (Sussman, 2012). The first international pilot study completed was in Wuhan, China (Zheng et al., 2004), with an intent-to-treat 30-day quit rate of 11% at a four-month follow-up in adolescents aged 16–17 years. The second international pilot study was in Bashkortostan (Russia), with adolescents aged from 13 to 19 years. Intent-to-treat 30-day quit rate was 7.5% in the program group versus 0.1% in the control group at a six-month follow-up (Idrisov et al., 2013). In Spain, Project EX effectiveness has also shown a tobacco cessation in adolescents aged 13–19 years old, with a significantly higher 30-day intent-to-treat rate for adolescents who received the program (4.9%) compared to the control group (0%) (McCuller et al., 2006; Espada et al., 2015, 2016). Higher level of motivation to quit smoking was related to higher smoking quit rates; therefore, motivation to quit smoking is considered a mediator of the effects of the intervention.

Mediation analysis are especially useful to know how interventions work by identifying the variables that have the greatest influence on the effectiveness, and what other variables (on which the intervention has no impact) are particularly relevant to achieve the goal of the intervention, and they need to be revised to increase the effectiveness of the intervention (MacKinnon, 2008). McCuller et al. (2006) analyzed the role of motivation to quit smoking as a mediator variable of Project EX; however, more evidence is needed on what mechanisms are underlying this intervention’s effects. This longitudinal study aims to test the ND as a mediator of Project EX’s effectiveness to reduce tobacco consumption in adolescents from Spain.

## Materials and Methods

### School Recruitment and Experimental Design

The study was approved by the institutional review board at Miguel Hernandez University, Spain. The education authorities were informed of the study goals, and authorization was requested by the parents, who were informed by letter and requested to provide written consent for their children to participate in the study. The written parental consent was provided to all minors participating.

We contacted a convenience sample of 45 schools from 17 towns in the Alicante, a province of Spain. A first meeting with the school boards was held to present the objectives of the intervention, and a total of six high schools from three cities [Elche (*n* = 4), Crevillente (*n* = 1), and San Vicente (*n* = 1)], agreed to participate. The schools recruited were randomly assigned to one of two experimental conditions: treatment or standard care (control).

A total of seventeen Spanish graduate students were interested in implementing the program. A researcher who was previously trained by the program developer provided training to all persons who finally delivered the program.

Two translators were responsible for the translation into Spanish of the original version of the Project EX program content. The final version was revised by two bilingual researchers working at the Miguel Hernandez University by comparing the English and Spanish versions. Before implementing the program, the Spanish version of Project EX was assessed in a focus group (*n* = 10 high school students). This evaluation was helpful to test the feasibility of the program content and verified that it was clearly understood and culturally appropriate. In addition to language adaptations, some changes were made in the Project EX program to adapt it to Spanish culture. The Project EX classroom program is closely adapted from the clinic program (Sussman et al., 2001; Sun et al., 2007). The learning activities included strategies to quit smoking and learning skills for maintenance without smoking, with an interactive methodology based on motivation. The methodology of Project EX in Spain can be found somewhere else (Espada et al., 2015). The sessions and the objectives of the program are shown in **Table 1**.

**Table 1:** Project EX sessions and objectives.

Session name	Contents
Orientation	Imparts the ground rules for the group and discusses reasons for quitting tobacco.
Tobacco affects your life	Discusses how tobacco use can cause, rather than relieve stress.
Health dangers of tobacco use	Discusses the harmful substances in tobacco and how it can injure one’s body.
Quitting step 1-Making a commitment about not using tobacco	Discusses addiction to tobacco. Methods of quitting smoking, and physical and psychological aspects of withdrawal are discussed.
Quitting step 2-Managing withdrawal symptoms	Discusses more about nicotine, addiction, and strategies of avoiding addiction or managing withdrawal symptoms. Psychological coping includes self-forgiveness and avoiding false expectations regarding how quitting will and will not affect one’s life.
Taking care of a healthy body	Involves learning lifestyle balance strategies, including weight control and practicing a “yoga activity.”
Taking care of your piece of mind	Involves learning more coping strategies, including assertiveness training and anger management. Participants also learn the “letting feelings pass” meditation activity.
Not smoking again: commitment and avoiding relapse	Involves learning means to avoid using tobacco, and mentions how topics covered could be applicable to other substances.

### Participants

A total of 1,546 scholars aged 14–21 years old (mean age: 15.28; *SD* = 1.20; 46% were women) were evaluated by self-administered tests. Adolescents who were smokers (*N* = 501; 32%) were selected for this study. **Table 2** shows descriptive information about the sample.

**Table 2:** Baseline characteristics and reports of tobacco consumption measures at 12-month follow-up of participating Spanish adolescents by experimental condition.

Characteristics	Intervention group	Control group	Total	Test statistics^a^
Sample size (*N*)	240	261	501	
No. (*%*) male	114/240 (47.5)	132/261 (50.6)	246/501 (49.1)	0.473
Mean age (years) (*SD*)	15.66 (1.12)	15.98 (1.44)	15.83 (1.30)	-2.703ˆ**
Nationality				
Spanish	214/240 (89.2)	235/261 (90)	449/501 (89.6)	0.102
Other	26/240 (10.8)	26/261 (10)	52/501 (10.4)	
Live with… (%)				
Both parents	163/216 (75.5)	208/251 (82.9)	371/467 (79.4)	5.553
Mother	47/216 (21.8)	34/251 (13.5)	81/467 (17.3)	
Father	6/216 (2.8)	9/251 (3.6)	15/467 (15)	
School achievement (number of failed courses/per year)	3.37 (3.82)	2.88 (3.36)	3.11 (3.59)	1.476
Age of first cigarette smoked	13.19 (2.03)	13.47 (1.75)	13.34 (1.89)	-1.698
Number of daily cigarettes	1.88 (3.70)	2.43 (4.37)	2.15 (4.05)	-1.443
Number of cigarettes smoked within the last 7 days	8.45 (18.31)	13.85 (31.67)	11.18 (26.03)	-2.228ˆ*
Number of cigarettes smoked in the last month	2.26 (4.65)	3.06 (5.21)	2.66 (4.95)	-1.721
Exhaled carbon monoxide (ECM) (CO) level	2.58 (2.80)	2.56 (3.06)	2.57 (2.93)	0.054
Nicotine dependence (ND)	29.32 (8.58)	28.82 (8.45)	29.06 (8.51)	0.645

*p ≤ 0.05; **p ≤ 0.01; *SD*, standard deviation; ^a^*T*-test was used for significance testing of continuous variables, and χ^2^ test was used for significance testing of categorical variables; Higher scores represent higher level of ND.

### Data Collection and Measures

Participants were evaluated at pretest, posttest, and 12-month follow-up using paper-and-pencil questionnaire. Demographic variables included gender, age (years), nationality (born in Spain, or immigrated to Spain from another country), current living situation (with parents, live alone, other situation), and parents’ education (mean response across father’s (or stepfather’s) and mother’s (or stepmother’s) educational levels based on categories derived from Hollingshead and Redlich (1958).

Smoking behavior was assessed with the fill-in-the blank items asking “How many cigarettes have you smoked in the last month (30 days)?” and “How many cigarettes have you smoked in the last week (7 days)?”, and the assessment-day smoking behavior was measured with the item: “Did you smoke tobacco today?” The 8-item modified Fagerstrom Tolerance Questionnaire (mFTQ) was used to measure the level of ND (Prokhorov et al., 1996, 2000; Idrisov et al., 2013). An example of the item is: “How soon after waking do you smoke your first cigarette?” The higher sum score indicates the higher the participant’s level of ND. Cronbach alpha for mFTQ in this sample was appropriate (α = 0.87). Expired CO was assessed using a breath CO monitor (Micro+ Smokerlyzer; Bedfont Technical Instruments, Kent, UK^1^, accessed April 19, 2014) at pretest, posttest and follow-up evaluations. This measure was valuable to validate self-reported assessment-day smoking.

### Data Analysis

Descriptive statistics were computed for sociodemographic variables and main outcomes of the present study. Baseline differences between the control and experimental groups were calculated using *T*-test (quantitative variables) and Chi-square (χ^2^) (qualitative variables). Statistical analyses were carried out using SPSS Statistics v23.0.

**FIGURE 1:**
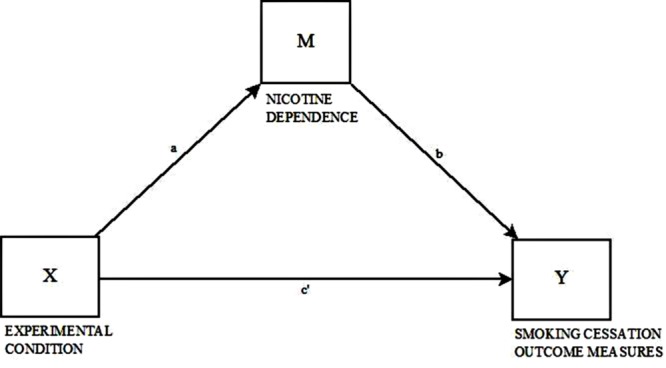
**Path diagram of the effect of the intervention on smoking cessation outcome measures at 12-month follow-up (Time 3) via nicotine dependence (ND) (Time 2), after controlling for baseline differences, gender, age, and school.** X, Independent variable; Y, Dependent variable; M, Mediator; a, b, c′, Regression coefficients. The 95% CIs for indirect effects were obtained by bootstrapping with 5,000 resamples.

Mediation analyses were implemented with the SPSS PROCESS macro (Hayes, 2013). We used 5,000 samples for bias-corrected bootstrap confidence intervals; and the significant criterion was *p* ≤ 0.05. The single-mediator model described by Hayes (2013) was used (**Figure 1**). The predictor was a binary variable contrasting a tobacco-use cessation program (Project EX) with the control group (non-intervention). Primary outcomes were continuous variables: three self-reported measures - number of cigarettes smoked in the last month, number of cigarettes smoked within the last 7 days, and number of daily cigarettes -, and a biological measure (exhaled CO level). Analyses were controlled for sex, age, school, and baseline measures.

We assigned the mediator as changes in the level of ND. Indirect effect is estimated in simple mediation models as a product of regression weight linking X–Y through M (Ind 1) (Hayes, 2013). Mediation analyses were conducted for the four primary outcomes using a product-of-coefficient approach (Preacher and Hayes, 2008). The effect of the intervention on the level of ND (M) is represented in the path α; while the effect of the level of ND (M) on each primary outcome (Y) is represented in the path β. If indirect effects do not include zero, there is a significant mediation.

## Results

**Table 3** shows descriptive statistics of the main outcomes and the mediator in each evaluation: baseline, posttest, and 12-month follow-up for the intervention and control groups. The program had a significant impact on the level of ND (M) (path α) as shown in **Table 4**. This finding shows that adolescents involved in the intervention group informed lesser level of ND at posttest than those in the control group.

**Table 3:** Self-report of tobacco consumption measures, and ND (as mediator) by condition and assessment period.

	Baseline		Posttest		12-month Follow-up	
	Intervention	Control	Intervention	Control	Intervention	Control
	(*N* = 240)	(*N* = 261)	(*N* = 149)	(*N* = 196)	(*N* = 139)	(*N* = 113)
Potential mediator mean (±) SD						
mFTQ ND^a^	26.15 (4.59)	25.75 (4.78)	25.56 (5.45)	26.42 (5.02)	26.48 (5.30)	28.44 (3.58)
Outcomes mean (±) SD						
Number of cigarettes smoked in the last month	2.26 (4.65)	3.06 (5.21)	1.50 (2.95)	2.57 (3.79)	1.98 (3.67)	2.83 (4.22)
Number of cigarettes smoked within the last 7 days	8.45 (18.31)	13.85 (31.67)	5.22 (13.57)	14.10 (56.14)	7.34 (18.29)	15.91 (31.90)
Number of daily cigarettes	1.88 (3.70)	2.43 (4.37)	1.42 (3.86)	2.93 (9.82)	1.14 (2.68)	2.47 (4.76)
Exhaled carbon monoxide (CO) level	2.58 (2.80)	2.56 (3.06)	1.97 (2.22)	2.56 (2.91)	1.58 (1.27)	2.14 (2.10)

**SD*, standard deviation. ^a^Modified Fagerstrom Tolerance Questionnaire score.*

**Table 4:** Nicotine dependence as a mediator of the effect of Project EX, compared with a control group, on tobacco use measures by the 12-month follow-up among adolescents from Spain.

Main outcome (*Y*)	Effect of the intervention (*X*) on the ND (*M*)^a^			Effect of ND as a mediator (M) on the main outcome (*Y*)^b^			Indirect effect of ND as the mediator on the main outcome (*Y*)
	α Path (SE)	95% CI	*p*-value	β Path (SE)	95% CI	*p*-value	Ind 1^c^ [ACI]^d^
Model 1	–2.43 (1.20)	–4.83, –0.04	0.046	3.59 (1.27)	1.06,6.13	0.0059	–8.78 [–22.44, –1.45]
Model 2	–2.93 (1.20)	–5.32, –0.54	0.016	2.07 (0.71)	0.65,3.50	0.0048	–6.09 [–14.09, –1.65]
Model 3	–2.93 (1.18)	–5.28, –0.58	0.015	0.30 (0.11)	0.08,0.52	0.0076	–0.89 [–2.07, –0.25]
Model 4	–3.69 (1.37)	–6.44, –0.94	0.009	0.14 (0.05)	0.02,0.26	0.0163	–0.53 [–1.29, –0.15]

*^a^The α path is the Project EX’s effect on each potential mediator. ^b^The β path is the effect of mFTQ ND mediator on the main outcome (*Y*). ^c^Ind 1 = *X* – *M*1 – *Y*. ^d^Asymmetric confidence interval based on bootstrap method with 5,000 replicates. The mediation analyses were adjusted for baseline differences between Project EX and the control group, gender, age, baseline value of the mediator and school. Model 1: Main outcome (*Y*) = Number of cigarettes smoked in the last month. Model 2: Main outcome (*Y*) = Number of cigarettes smoked within the last 7 days. Model 3: Main outcome (*Y*) = Number of daily cigarettes. Model 4: Main outcome (*Y*) = Exhaled carbon monoxide (CO) level.*

Path β shows the existing significant relationship between the level of ND and all the main outcomes: number of cigarettes smoked in the last month (*p* = 0.04), number of cigarettes smoked within the last 7 days (*p* = 0.01); number of daily cigarettes (*p* = 0.01), and exhaled carbon monoxide (ECM) (CO) level (*p* = 0.009). In all the analyzed models, path β shows that the higher level of ND (posttest) was related to higher number of cigarettes smoked in the last month, higher number of cigarettes smoked within the last 7 days, higher number of daily cigarettes, and higher level of ECM (CO) level at 12-month follow-up.

The intervention positively reduced the number of cigarettes smoked in the last month (ACI = –22.44, –1.14), number of cigarettes smoked within the last 7 days (ACI = –14.09, –1.65); number of daily cigarettes (ACI = –2.07, –0.25), and ECM (CO) level (ACI = –1.29, –0.14) after 12-month period indirectly through reducing the level of ND.

## Discussion

This work’s objective was to test the ND as mediator of Project EX’s effect to reduce tobacco consumption in adolescents from Spain. Findings show that the intervention had a significant impact on the level of ND short-time, and this variable was a mediator of the intervention’s effect on several tobacco use measures. Compared to the control group, adolescents who received Project EX reduced their level of ND short-term, and they were more likely to report lesser tobacco use at 12-month follow-up, in terms of number of cigarettes smoked in the last month, number of cigarettes smoked within the last 7 days, number of daily cigarettes, and ECM level. In the present study, there was a significant relationship between the level of ND and all these main outcomes. The results are consistent with other studies that suggest that lower ND corresponds to lower tobacco consumption (González et al., 2016; Ruiz and Miranda, 2016).

Previously, McCuller et al. (2006) evaluated the effects of Project EX on changing motivation to quit smoking. It concludes that motivation to quit smoking is a plausible mediator of cessation program effects since higher level of motivation was statistically significantly related to higher smoking quit rates. In the present study, composed of Spanish adolescents, motivation was discarded as a mediator of the intervention’s effects to reduce tobacco consumption because of the characteristics of the sample. At one-year follow-up of the smoking intervention program with Spanish adolescents there was a lack of general readiness to be involved in cessation programming. Evidence of this were the low percentage of schools that agreed to be involved initially, and the groups of young smokers that dropped out before the program started or after the first session (39.3%), as well as a high attrition rate. This results may be explained by the fact of the implementation was held after school, and students did not receive any incentive for participating in the present study (Espada et al., 2016). This suggests that participants were not highly motivated to participate in the program.

In Spain, Project EX is the only school-based tobacco cessation program whose effects have been proven to be positive to reduce tobacco consumption at 6 and 12 months (Espada et al., 2015, 2016). Furthermore, the assessment of expired CO by use of a breath CO monitor validates self-reported smoking responses. It is noteworthy that results were similar when the main outcome was assessed with self-report measures than when biological measures were used. Although ND was a mediator of the intervention’s effects on every main outcome, it is curious that the coefficient of mediation was higher in the model with cigarette consumption in the last month as a main outcome compared to the rest of measures. The coefficient of mediation decreased gradually as the main outcome implied a more limited time or more recent use of tobacco (last 7 days, daily). This effect may be explained by memory effect of the participants; in other words, it may be more easily remembered the number of daily cigarettes than the number of cigarettes smoked in the last month. Furthermore, it is important to note that the measurement of CO is a biological measure, and therefore, it is more accurate.

The results of this study have important implications for the tobacco cessation in Spanish adolescents. The results permit identifying the mechanisms involved in the effectiveness of a tobacco cessation program 12 months subsequent to its application. The present study has at least four strengths. This is the first Spanish study that explores ND as a mediator of the long-term efficacy of Project EX to reduce tobacco consumption in adolescents. A considerable sample size was used to explore this issue. The longitudinal design (including 12-month follow-up) is an important strength of the present study since there is a lack of this type of studies in prevention science. A biological measure was used, rather than only self-reported measures, which provides a more direct indicator of tobacco consumption in this population.

Nevertheless, the present study has some limitations. First, although the study involved a large sample, it is not from a varied geographical origin, so it is necessary to expand this study to other regions of the country. Second, U.S. and international survey data reveal that youth are aware of e-cigarettes and use of these products in this population is rapidly increasing (Durmowicz, 2014), and currently unregulated (Dutra and Glantz, 2014). In this study conventional tobacco consumption is evaluated, so it could be that adolescent consumers of e-cigarettes do not identify as tobacco consumers. Future studies on the consumption of tobacco should consider the use of this popular type of electronic nicotine delivery system.

Despite the limitations, this is the first Spanish study that explores ND as a mediator of the long-term efficacy of Project EX to reduce tobacco consumption in adolescents. However, more research is required for a better understanding of the success and/or failure of smoking cessation and prevention programs in adolescent population.

## Author Contributions

All individuals listed as authors have: Contributed substantially to the conception and design of the work. Drafted the work or revised it critically for important intellectual content. Have given final approval of the version to be published. Agree to be accountable for all aspects of the work in ensuring that questions related to the accuracy or integrity of any part of the work are appropriately investigated and resolved.

## Conflict of Interest Statement

The authors declare that the research was conducted in the absence of any commercial or financial relationships that could be construed as a potential conflict of interest.

The reviewer SW and handling Editor declared their shared affiliation, and the handling Editor states that the process nevertheless met the standards of a fair and objective review.

## Footnotes

1 http://www.bedfont.com/ch/smokerlyzer/micro
